# Efficacy and safety of Chinese medicine combined with acupuncture in the treatment of chronic urticaria: A meta-analysis

**DOI:** 10.1097/MD.0000000000030381

**Published:** 2022-09-09

**Authors:** Zhenxiong Lu, Qiujun Zhou, Shiqian Chai, Huifeng Yang, Jinhui Wang, Hongbin Luo, Yi Cao, Maocan Tao

**Affiliations:** a Yongkang Maternal and Child Health Care Hospita, Zhejiang Province, Jinhua, China; b Department of First Clinical Medical College, Zhejiang Chinese Medical University, Hangzhou, Hangzhou, China 310000; c Wenzhou City Hospital of Traditional Chinese Medicine and Western Medicine Combined, Wenzhou, China; d Department of Dermatology, The First Affiliated Hospital of Zhejiang Chinese Medical University, Hangzhou, China.

**Keywords:** acupuncture, Chinese medicine, chronic urticaria, efficacy and safety, meta-analysis

## Abstract

Increasing studies have shown that Chinese medicine combined with acupuncture has a significant effect on chronic urticaria, which can treat both symptoms and root causes. This meta-analysis aims to compare the efficacy and safety differences between acupuncture combined with conventional Western medicine, so as to provide guidance for the clinical treatment of chronic urticaria.

**Methods:**

We searched Pubmed, Embase, Cochrane Library, CNKI, Wanfang, CQVIP, and CBM from the establishment of the database to August 2021. We included randomized controlled trial study that the experimental group was acupuncture combined with traditional Chinese medicine, while the control group was treated with conventional Western medicine. We excluded repeated publication, researches without full text, incomplete information, or inability to conduct data extraction and animal experiments, reviews, and systematic reviews. STATA 15.1 was used to analyze the data.

**Results:**

The pooled results show that total effective rate of acupuncture combined with traditional Chinese medicine group was significantly higher than that in the conventional Western medicine group (ratio rate [RR] = 1.29, 95% confidence interval [CI]: 1.2–1.38). Additionally, the pooled results show that Urticaria Activity Score (standardized mean difference = –1.51, 95% CI: –2.24 to –0.78) and pruritus score (standardized mean difference = –1.09, 95% CI: –1.71 to –0.47) of acupuncture combined with traditional Chinese medicine group was significantly lower than that in conventional Western medicine group, while there is no significant difference in wheal score between acupuncture combined with traditional Chinese medicine group and conventional Western medicine group. Importantly, the pooled results show that recurrence rate (RR = 0.35, 95% CI: 0.19–0.64) and the incidence of adverse events (RR = 0.27, 95% CI: 0.10–0.75) of acupuncture combined with traditional Chinese medicine group were all significantly lower than that in conventional Western medicine group.

**Conclusion:**

Our research results found that traditional Chinese medicine combined with acupuncture has a more significant effect than conventional Western medicine and can significantly reduce the recurrence rate and the incidence of adverse reactions. The application of traditional Chinese medicine combined with acupuncture in the treatment of chronic urticaria should be further promoted.

## 1. Introduction

Urticaria is a common allergic skin disease. It is a kind of localized edema reactive disease caused by a common skin and mucosal small blood vessel dilation and osmotic increase caused by various reasons.^[1]^ Chronic urticaria is defined as episodic or daily hives lasting for at least 6 weeks and impairs quality of life.^[2]^ The clinical manifestation is that rubella lesions of varying sizes occur suddenly, disappear quickly, and the itching is severe, leaving no traces after healing. It belongs to the categories of “rubella” and “rubella addiction” in Chinese medicine. This disease can be seen in any age group and season, and 15% to 20% of people have had this disease at least once in their lives.^[3]^

At present, the treatment of chronic urticaria in Western medicine is mainly through long-term oral administration of antihistamines such as cetirizine hydrochloride and desloratadine citrate.^[4]^ However, simple Western medicine desensitization treatment often has adverse events such as fatigue and lethargy, strong drug resistance and high recurrence rate after stopping the drug.^[5]^ Therefore, in recent years, more and more researches have begun to explore new treatment modes and improve diagnosis and treatment measures. Traditional Chinese medicine (TCM) treatments such as TCM preparations and acupuncture, cupping, and other characteristic therapies have been integrated into the clinic. Many Chinese medicine preparations, such as Xiaofeng San, have the effects of clearing away heat, removing dampness and nourishing blood, and can effectively relieve the itching symptoms of urticaria.^[6]^ Acupuncture treatment can evacuate wind pathogens, replenish qi, and nourish blood, regulate the yin and yang of the internal organs, strengthen the body’s immunity to defend against the invasion of wind pathogens, and fundamentally improve the prevention and treatment of urticaria.^[7]^ Acupuncture belongs to the external treatment of TCM, through stimulating acupoints, to achieve the adjustment of zang and fu, nourishing and dispelling evil, according to the theory of TCM, acupuncture treatment of skin diseases is to enhance the resistance of the human body by adjusting defensive Qi. Commonly used for the treatment of chronic urticaria acupoints fengchi, Quchi, Blood sea, Shu, Sanyinjiao, Zusanli, and other points, with qi line blood, dispel wind, and relieve itching, in line with the principle of chronic urticaria treatment, so as to achieve the purpose of treating the disease.^[7]^ Increasing studies have shown that Chinese medicine combined with acupuncture has a significant effect on chronic urticaria, which can treat both symptoms and root causes.^[8]^ Currently, there is a lack of systematic evidence-based medical evidence on the efficacy and safety of Chinese medicine combined with acupuncture in the treatment of chronic urticaria. Therefore, this article aims to conduct a meta-analysis of the literature on the treatment of chronic urticaria with TCM combined with acupuncture, and compare the efficacy and safety differences between acupuncture combined with conventional Western medicine, so as to provide guidance for the clinical treatment of chronic urticaria.

## 2. Methods

### 2.1. Literature inclusion and exclusion criteria

Inclusion criteria: the study type is a randomized controlled trial study; the experimental group was acupuncture combined with TCM, while the control group was treated with conventional Western medicine; the language is limited to Chinese and English.

Exclusion criteria: repeated publication; research without full text, incomplete information or inability to conduct data extraction; animal experiments; reviews and systematic reviews.

### 2.2. Search strategy

In this meta-analysis, we searched Pubmed, Embase, Cochrane Library, CNKI, Wanfang, CQVIP, and CBM from establishment of the database to August2021. The Chinese search terms are mainly: “Acupuncture” “Chronic urticaria”. The English search terms are as follows: “Acupuncture”, “Pharmacopuncture”, “Chronic urticaria”, “Chronic Inducible Urticaria”, “Chronic Spontaneous Urticaria”, and “Idiopathic Chronic Urticaria”.

### 2.3. Literature screening and data extraction

&&The literature search, screening, and information extraction were all independently completed by 2 researchers. When there were doubts or disagreements, the decision was made after discussion or consultation with a third person. The data extraction included the author, year, research type, number of cases, gender, age of patients, duration of disease, measures, and the indicators for evaluating outcome, including total effective rate, Urticaria Activity Score (UAS), wheal score, pruritus score, recurrence rate, and the incidence of adverse events.

### 2.4. Literature quality assessment

Two researchers independently carried out the literature quality evaluation, using the Review manager 5.3 software risk assessment tool, according to the Cochrane risk assessment scale, according to the random sequence generation, allocation hiding, blinding, whether the research results are blindly evaluated, and the result data are complete evaluate the included literature based on gender, choice of report research results, other biases, etc, and decide through discussion or consultation with a third party when opinions are inconsistent. This meta-analysis is performed based on the related items of the Preferred Reporting Items for Systematic Reviews and Meta-analysis statement.^[9]^

### 2.5. Data synthesis and statistical analysis

Review manager 5.3 was used to analyze the data. Ratio rate (RR) (95% confidence interval [CI]) was used to combine the binary variable, and standardized mean difference (SMD) (95% CI) was used to combine the continuous variable. *I*^2^ is used to evaluate heterogeneity. If the heterogeneity test is *P* ≥ .1 and *I*^2^ ≤ 50%, it indicates that there is homogeneity between studies, and the fixed effects model is used for combined analysis; if *P* < .1, *I*^2^ > 50%, it indicates that the study If there is heterogeneity, use sensitivity analysis to find the source of heterogeneity. If the heterogeneity is still large, use the random effects model or give up the combination of results and use descriptive analysis. Funnel plot and Egger test was used to analyze publication bias.

## 3. Results

### 3.1. The results of literature search

**Figure 1.**
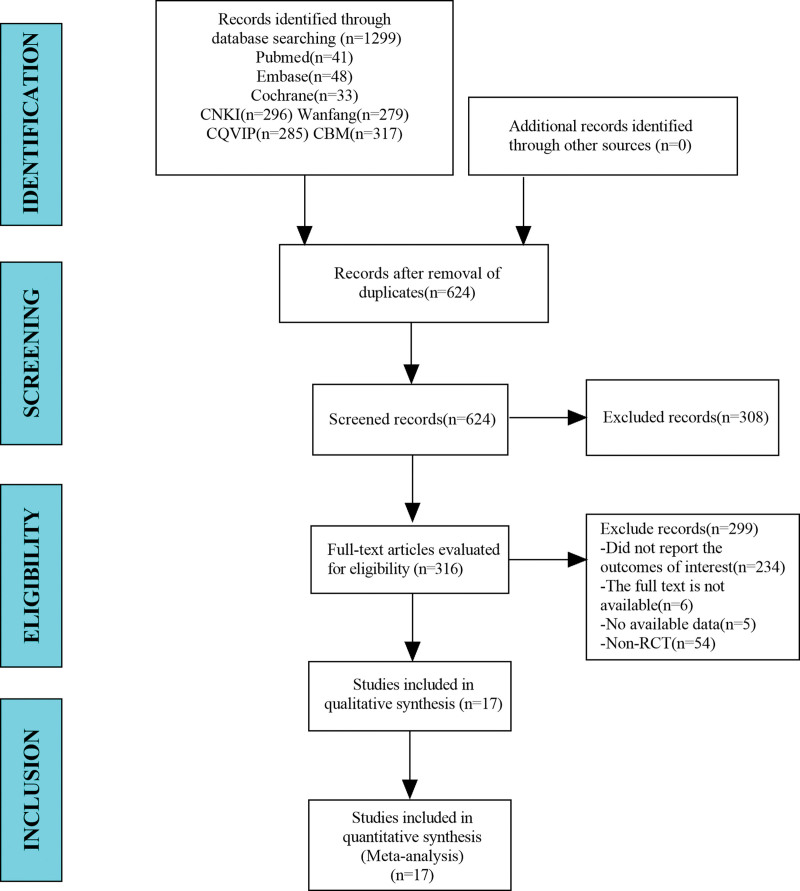
Flow diagram for selection of studies. RCT = randomized controlled trial.

In this study, a total of 1299 studies were retrieved from the database. After eliminating duplicate studies, 624 were obtained. After browsing titles and abstracts, 316 studies were obtained. Finally, 17 articles were included in the meta-analysis (Fig. 1).

### 3.2. Baseline characteristics and quality assessment of the included studies

#### 3.2.1. Baseline characteristics.

A total of 17 randomized controlled trial studies were included in this meta-analysis. The sample size of patients ranged from 60 to 120 and totaled 1220 patients, including 617 patients in the acupuncture combined with TCM group and 603 patients in the conventional Western medicine group. The main Chinese medicines used are Guizhi Decoction, Xiaofeng powder, Yangxue Xiaofeng Decoction, and so on and the main Western medicines used are cetirizine, loratadine, mizolastine, and so on (Table 1).

**Table 1:** Baseline characteristics of the included studies.

Author	Year	Research type	Number of cases		Gender (male/female)		Age		Duration of disease (yr)		Measures	
			Experimental group	Control group	Experimental group	Control group	Experimental group	Control group	Experimental group	Control group	Experimental group	Control group
Gou^[10]^	2019	RCT	30	30	16/14	17/13	44.66 ± 6.12	44.85 ± 6.62	2.78 ± 0.34	2.67 ± 0.54	Acupuncture + traditional Chinese medicine	Cetirizine
Zhang^[11]^	2013	RCT	30	30	12/18	14/16	33.6	34.8	2.3	2.7	Acupuncture + Guizhi Decoction	Loratadine
Chen^[12]^	2017	RCT	29	29	19/10	18/11	35.2 ± 0.9	34.5 ± 1.1	2.3 ± 1.2	2.2 ± 0.9	Acupuncture + Guizhi Decoction	Loratadine
Li^[13]^	2004	RCT	30	30	19/11	20/10	42.51 ± 10.42	42.71 ± 11.42	1–5	0.5–6	Acupuncture + Xiaofeng powder	Loratadine
Wang^[6]^	2018	RCT	50	50	21/29	23/27	34.87 ± 5.63	35.21 ± 5.41	2.11 ± 1.26	2.17 ± 1.28	Acupuncture + Xiaofeng powder	Loratadine
Ye^[14]^	2018	RCT	28	28	17/11	15/13	30.8 ± 2.6	31.2 ± 3.1	2.5 ± 0.5	2.2 ± 0.6	Acupuncture + Xiaofeng powder	Cetirizine
Zhou^[15]^	2015	RCT	60	60	35/25	35/25	35.8 ± 3.7	33.3 ± 5.7	1.5 ± 0.9	1.6 ± 0.1	Acupuncture + Xiaofeng powder	Cetirizine
Zhao^[16]^	2019	RCT	30	30	19/11	17/13	31.4 ± 3.3	31.4 ± 3.0	2.4 ± 0.7	2.1 ± 0.5	Acupuncture + Xiaofeng powder	Cetirizine
Xijin^[17]^	2016	RCT	13	13	5/8	3/10	45 ± 3.2	43 ± 3.5	2.5 ± 0.2	2.6 ± 0.5	Acupuncture + Xiaofeng powder	Loratadine
Hu^[18]^	2018	RCT	44	44	23/21	23/21	32.23 ± 2.82	34.45 ± 2.55	2.45 ± 1.26	2.57 ± 1.64	Acupuncture + Yangxue Xiaofeng Decoction	Mizolastine
Wang^[19]^	2010	RCT	44	38	–	–	31.4		0.71		Acupuncture + Yangxue Xiaofeng Decoction	Mizolastine
Yuan^[20]^	2014	RCT	32	32	17/15	13/19	31 ± 1.7	29 ± 2.2	6.4 ± 1.2	4.3 ± 2.6	Acupuncture + traditional Chinese medicine	Ebastine
Ye^[21]^	2007	RCT	48	40	20/28	16/24	32.3	31.7	–	–	Acupuncture + Xiaofeng powder	Chlorpheniramine
Qian^[22]^	2015	RCT	39	39	23/16	18/21	23 ± 4. 8	27 ± 2.9	4.6 ± 1.7	6.2 ± 1.6	Acupuncture + traditional Chinese medicine	Cetirizine
Sun^[23]^	2017	RCT	42	40	18/24	16/24	39.4 ± 15.3	38.5 ± 16.1	2.6 ± 1.4	2.7 ± 1.4	Acupuncture + Sangui Lizhong Wan	Loratadine
Liao^[24]^	2015	RCT	30	30	13/17	10/20	32.12 ± 9.58	30.07 ± 6.74	1.6 ± 1.0	2.0 ± 1.4	Acupuncture + traditional Chinese medicine	Loratadine
Xie^[25]^	2020	RCT	38	40	17/21	20/20	39.66 ± 11.86	43.70 ± 11.77	5.87 ± 4.19	7.13 ± 4.99	Acupuncture + Heying Qufeng Prescription	Tranilast

RCT = randomized controlled trial.

### 3.3.2. Quality assessment of the included studies.

**Figure 2.**
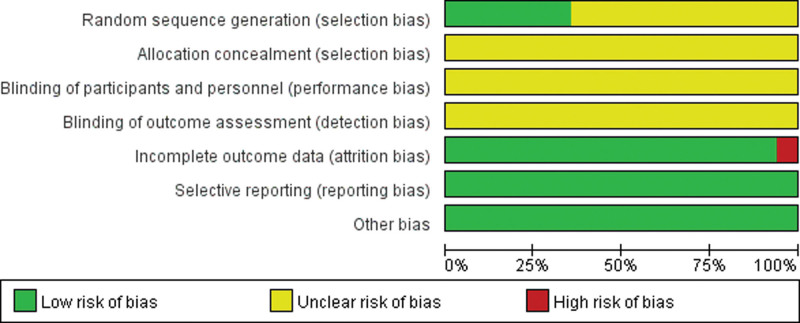
Risk of bias items.

**Figure 3.**
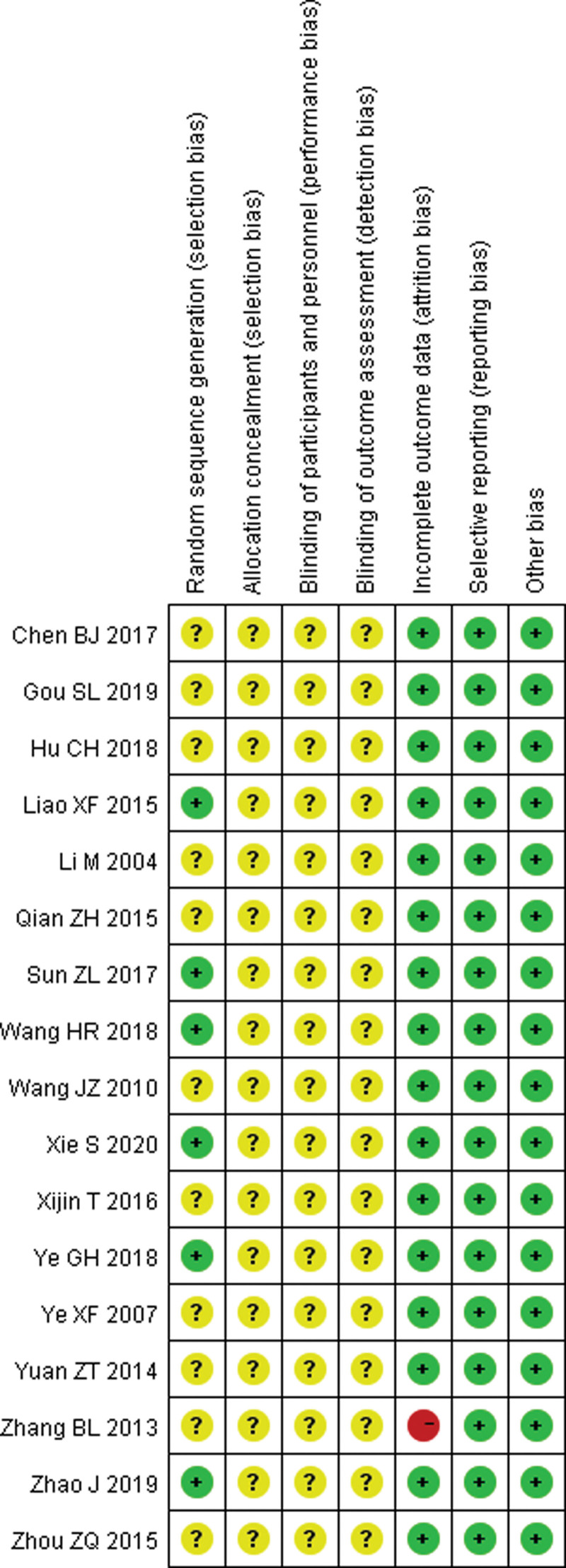
Risk of bias summary.

Methodological quality assessment was performed according to Preferred Reporting Items for Systematic Reviews and Meta-analysis guidelines. Six articles illustrate random methods, but none of all articles illustrate assignment concealment. Additionally, none use blind methods (Figs. 2–3).

### 3.3. Results of meta-analysis

**Figure 4.**
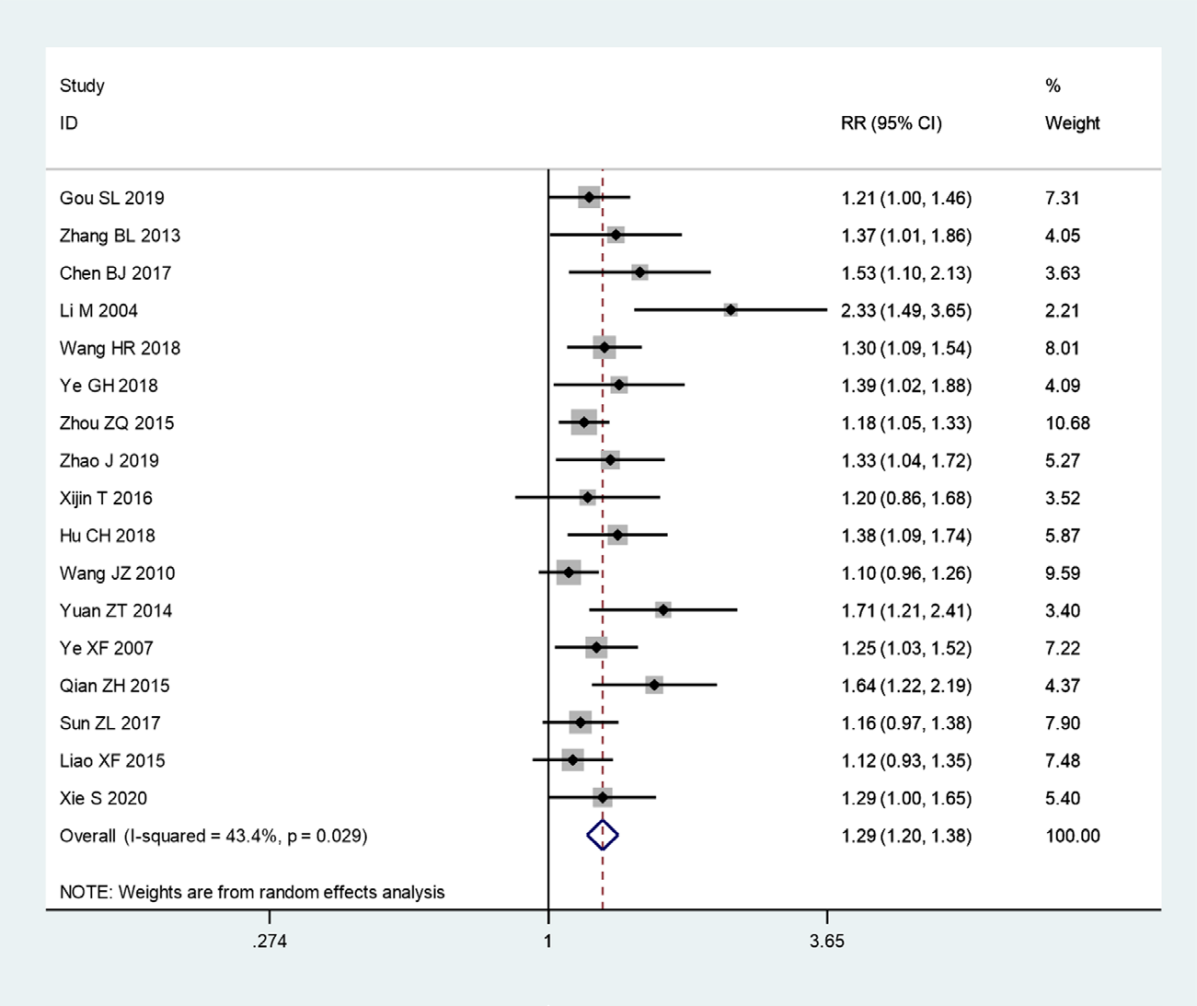
Total effective rate of acupuncture combined with traditional Chinese medicine group and conventional Western medicine group. CI = confidence interval, RR = ratio risk.

We first pooled the total effective rate of acupuncture combined with TCM group and conventional Western medicine group. Since there is significant heterogeneity (*I^2^ = *43.4%, *P = *.029), a meta-analysis was conducted through a random effects model. The pooled results show that total effective rate of acupuncture combined with TCM group was significantly higher than that in conventional Western medicine group (RR = 1.29, 95% CI: 1.2–1.38, *P* ≤ .01; Fig. 4).

**Figure 5.**
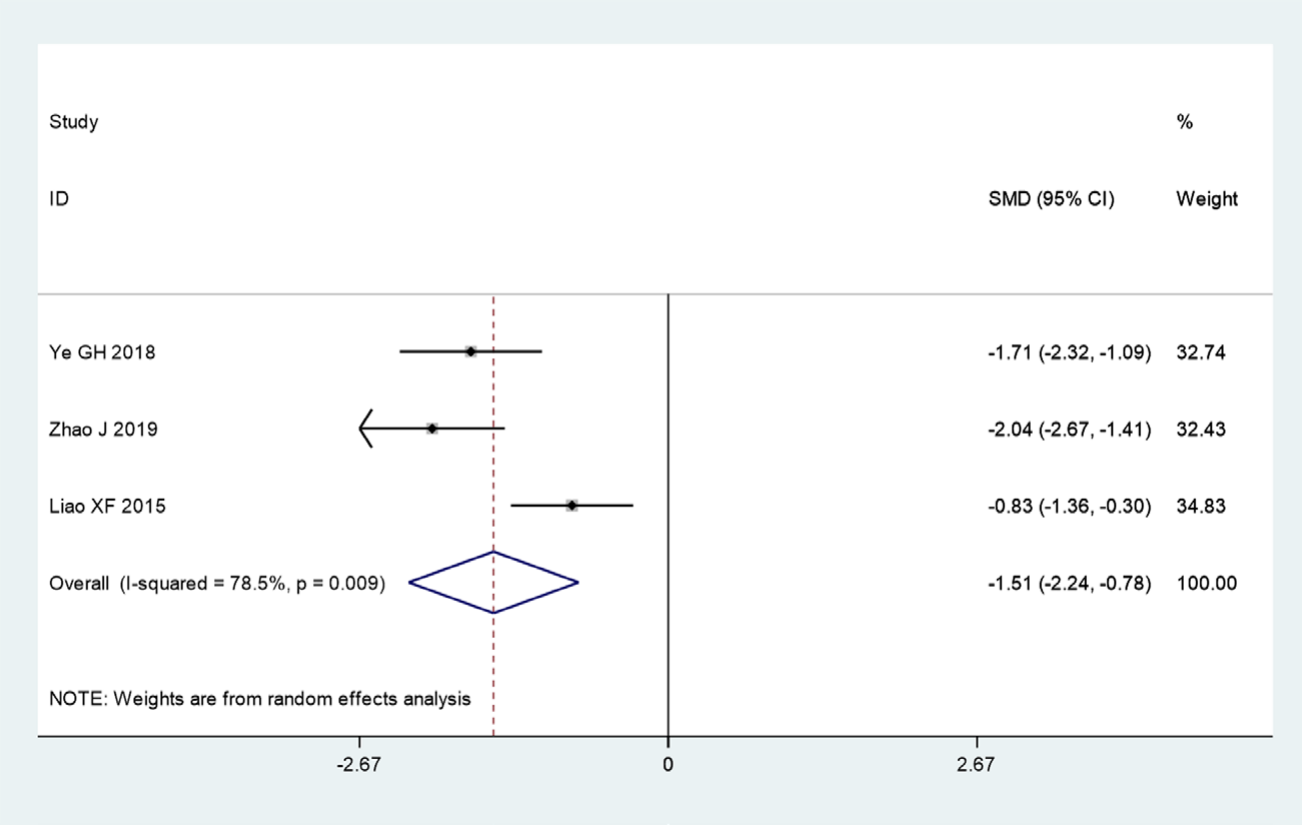
UAS of acupuncture combined with traditional Chinese medicine group and conventional Western medicine group. CI = confidence interval, SMD = standardized mean difference, UAS = Urticaria Activity Score.

**Figure 6.**
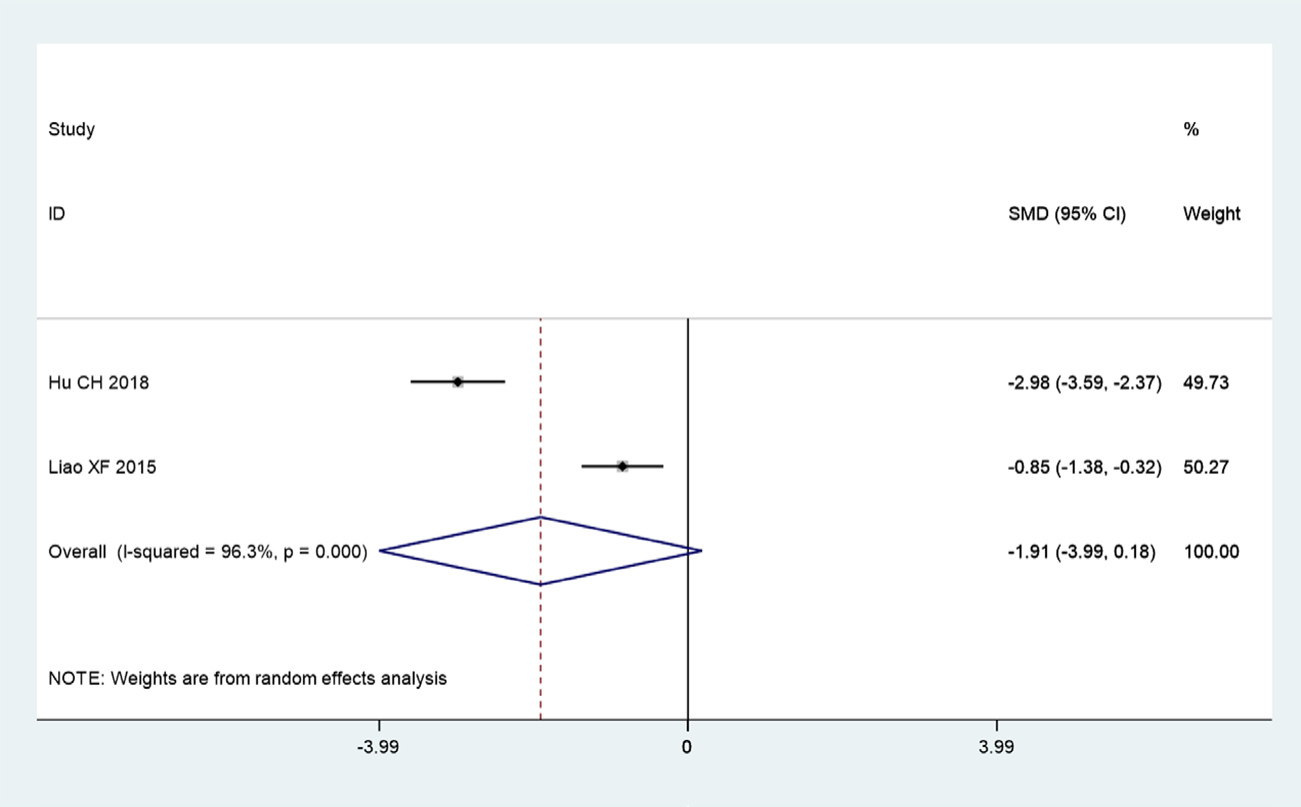
Wheal score of acupuncture combined with traditional Chinese medicine group and conventional Western medicine group. CI = confidence interval, SMD = standardized mean difference.

**Figure 7.**
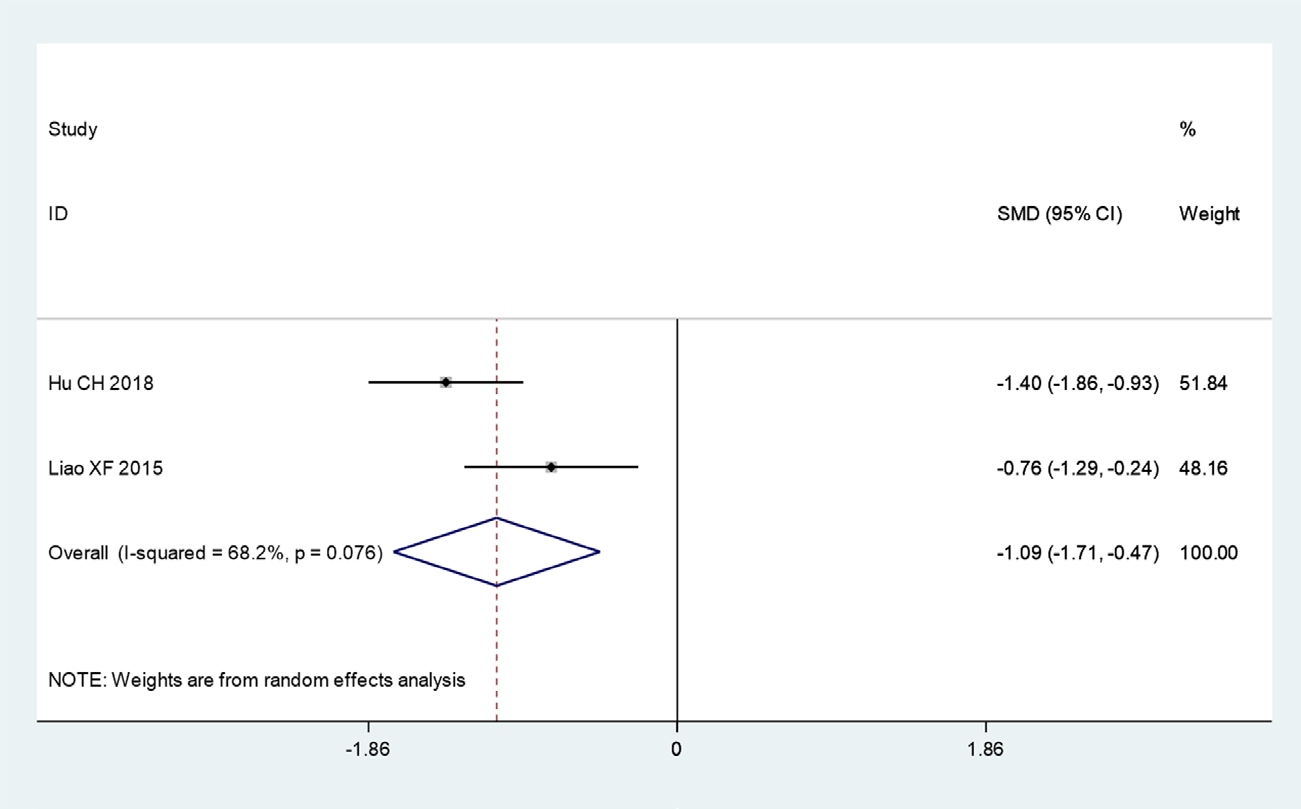
Pruritus score of acupuncture combined with traditional Chinese medicine group and conventional Western medicine group. CI = confidence interval, SMD = standardized mean difference.

Next, we analyzed the symptom score, including UAS, wheal score, and pruritus score of acupuncture combined with TCM group and conventional Western medicine group. The pooled results show that UAS (SMD = –1.51, 95% CI: –2.24 to –0.78, *P* ≤ .01; *I^2^ = *78.5%, *P = *.009; Fig. 5) and pruritus score (SMD = –1.09, 95% CI: –1.71 to –0.47, *P* = .001; *I^2^ = *68.2%, *P = *.076; Fig. 6) of acupuncture combined with TCM group was significantly lower than that in conventional Western medicine group. However, the pooled results show that there is no significant difference in wheal score between acupuncture combined with TCM group and conventional Western medicine group (SMD = –1.91, 95% CI: –3.99 to 0.18, *P* = .073; *I^2^ = *96.3%, *P ≤ *.01; Fig. 7).

**Figure 8.**
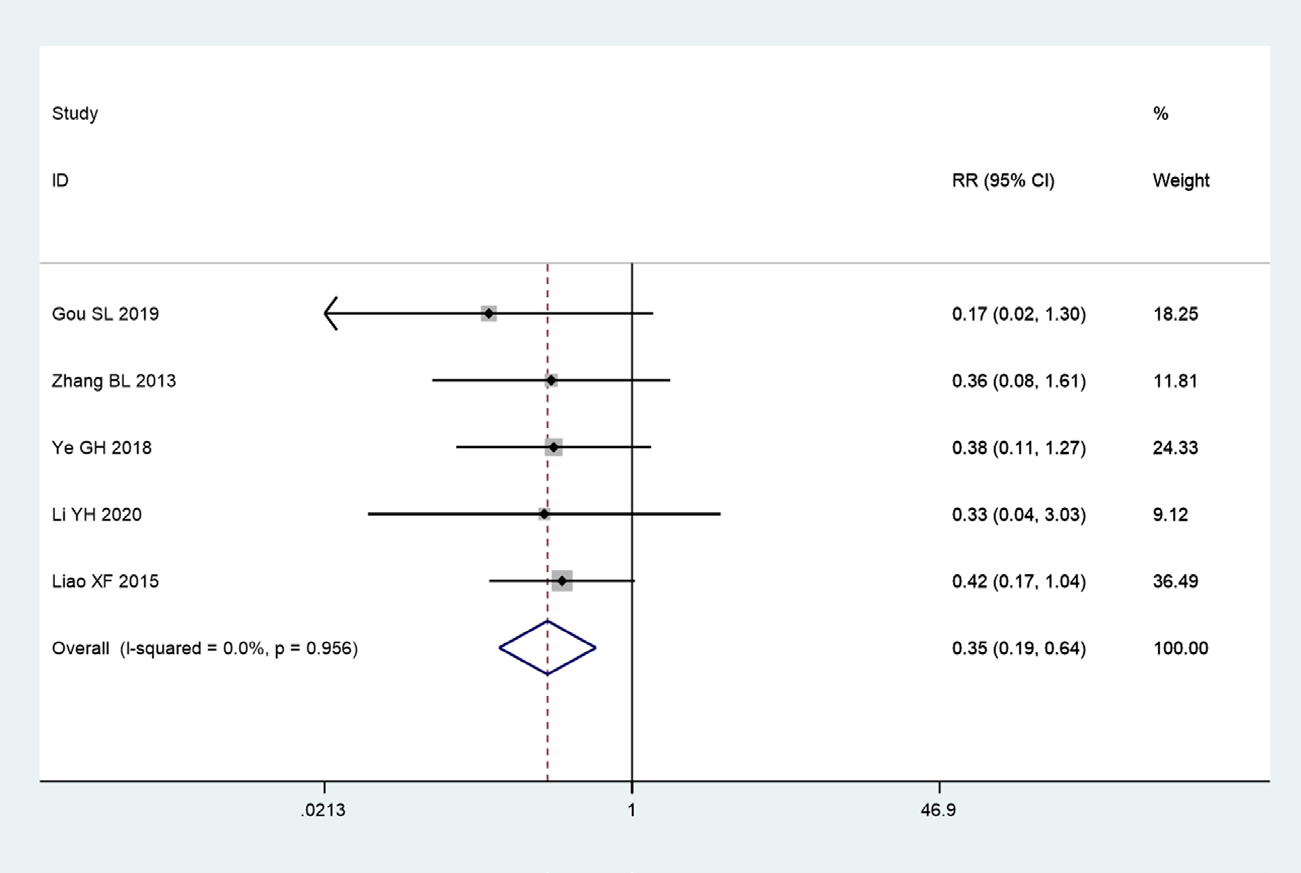
Recurrence rate of acupuncture combined with traditional Chinese medicine group and conventional Western medicine group. CI = confidence interval, RR = ratio risk.

**Figure 9.**
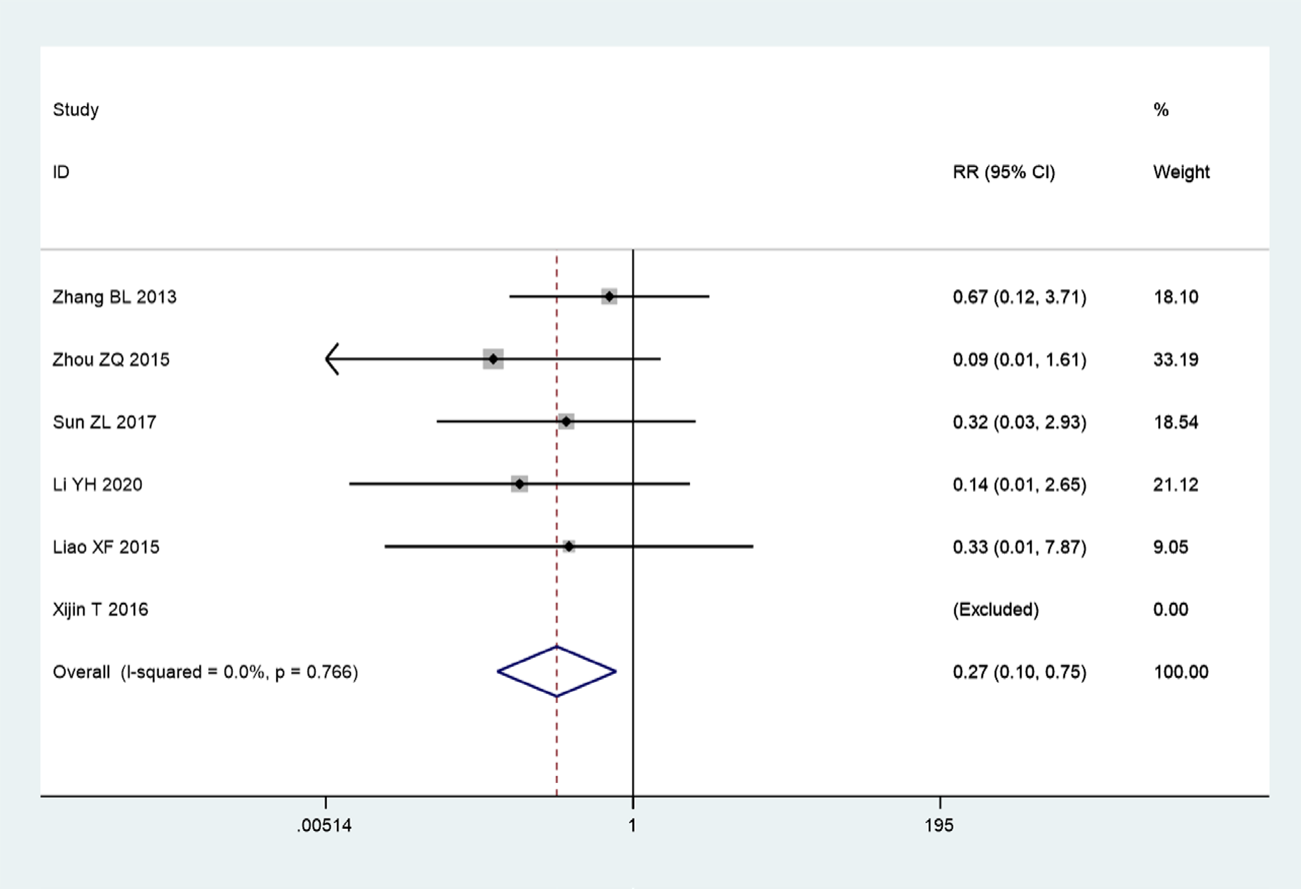
Incidence of adverse events of acupuncture combined with traditional Chinese medicine group and conventional Western medicine group. CI = confidence interval, RR = ratio risk.

Additionally, we explored the recurrence rate and incidence of adverse events of acupuncture combined with TCM group and conventional Western medicine group. Since there is no significant heterogeneity (*I^2^ = *0.0%, *P = *.956), a meta-analysis was conducted through a fixed effects model. The pooled results show that recurrence rate of acupuncture combined with TCM group was significantly lower than that in conventional Western medicine group (RR = 0.35, 95% CI: 0.19–0.64, *P* = .001; Fig. 8). Since there is no significant heterogeneity (*I^2^ = *0.0%, *P = *.766), a meta-analysis was conducted through a fixed effects model. The pooled results show that the incidence of adverse events of acupuncture combined with TCM group was significantly lower than that in conventional Western medicine group (RR = 0.27, 95% CI: 0.10–0.75, *P* = .012; Fig. 9).

### 3.4. Subgroup analysis

**Figure 10.**
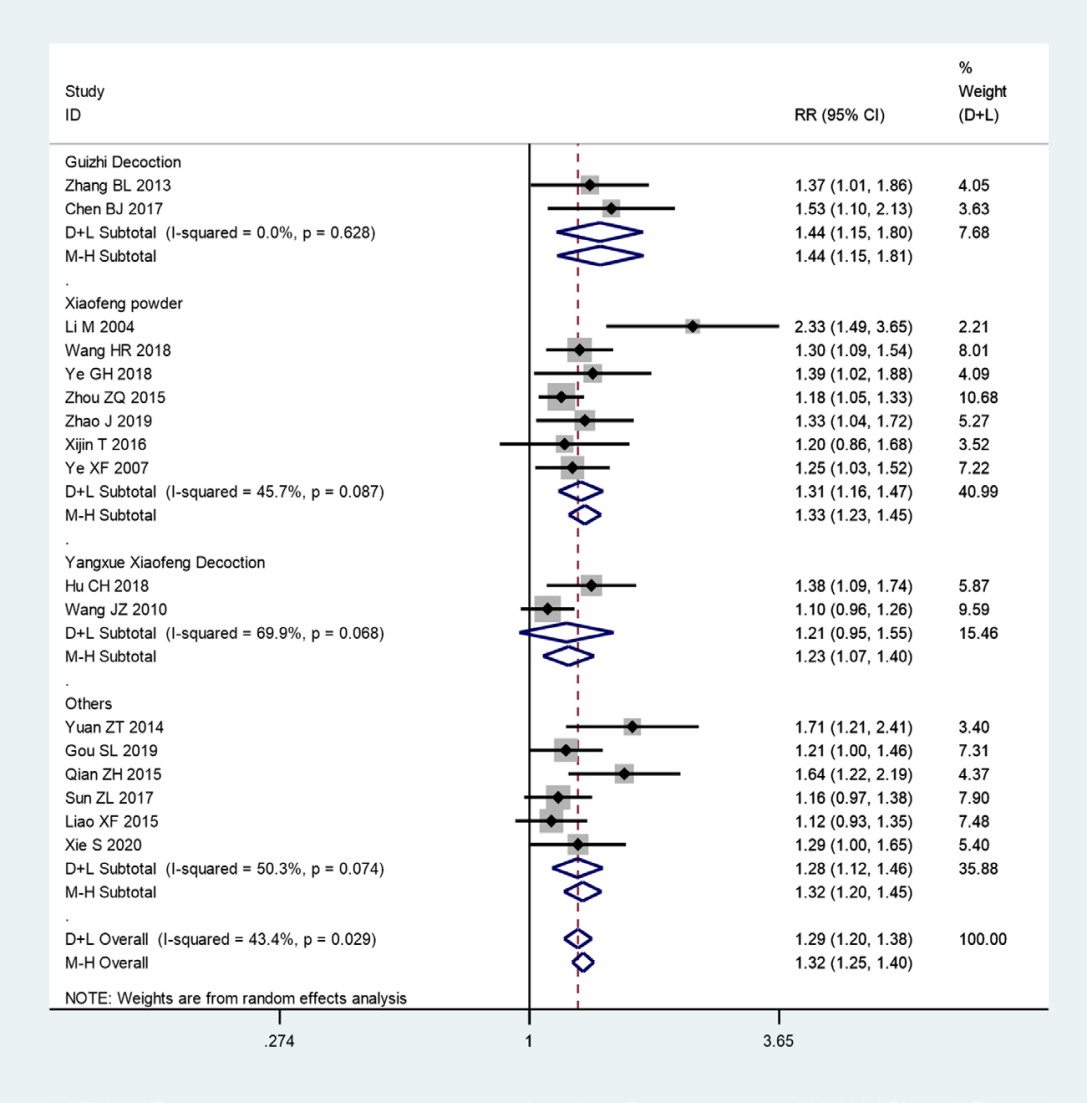
Subgroup analysis on the total effective rate according to different Chinese medicines. CI = confidence interval, RR = ratio risk.

**Figure 11.**
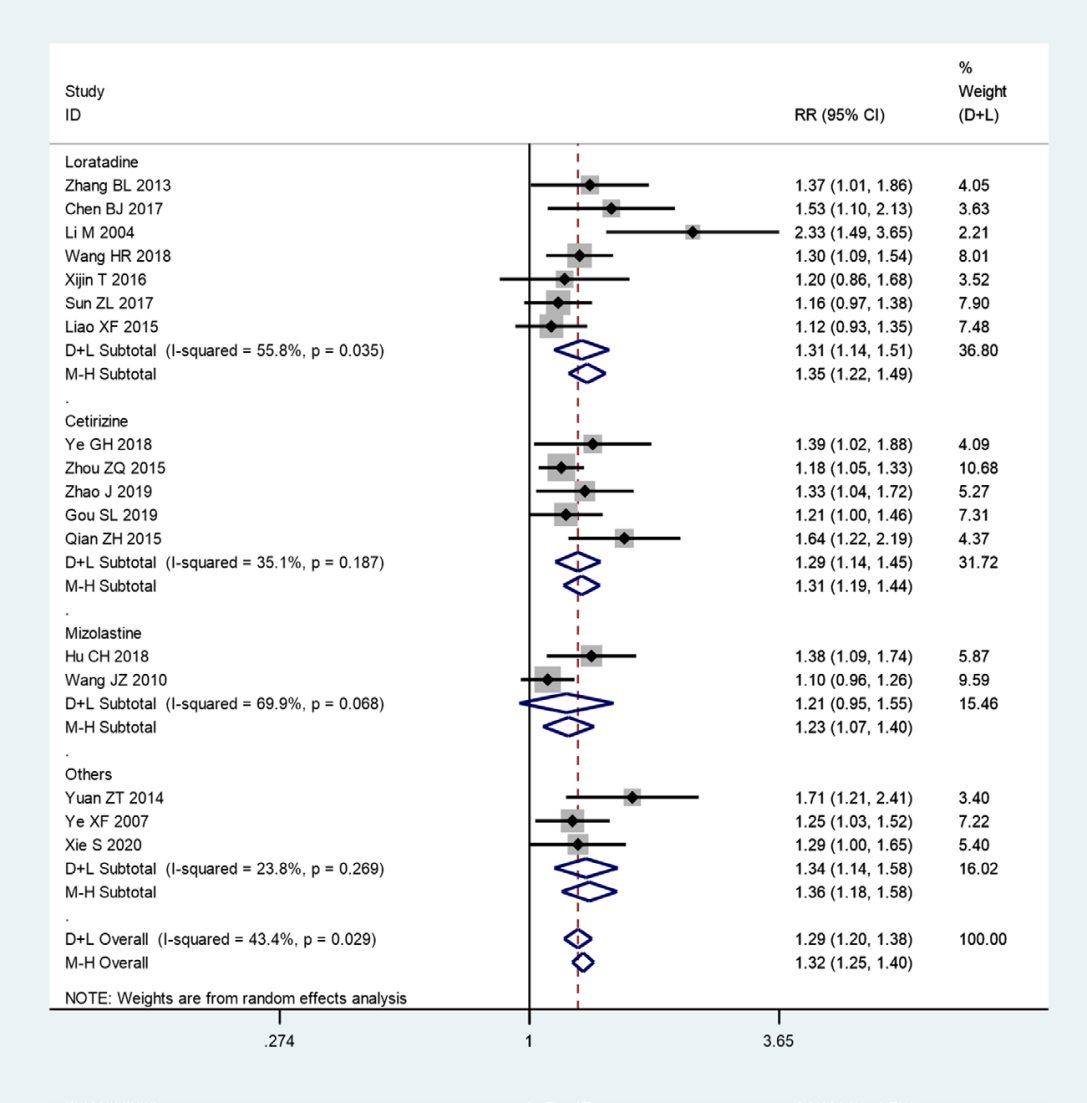
Subgroup analysis on the total effective rate according to different Western medicines. CI = confidence interval, RR = ratio risk.

Furthermore, we conducted subgroup analysis on the total effective rate according to different Chinese medicines and Western medicines, respectively. The pooled results show that the total effective rate of acupuncture combined with Guizhi Decoction (RR = 1.44, 95% CI: 1.15–1.81, *P* = .001; *I^2^ = *0.0%, *P = *.628) or Xiaofeng powder (RR = 1.31, 95% CI: 1.16–1.47, *P* ≤ .01; *I^2^ = *45.7%, *P = *.087) group was significantly higher than that in conventional Western medicine group (Fig. 10). However, the pooled results show that there is no significant difference between acupuncture combined with Yangxue Xiaofeng Decoction group and conventional Western medicine group (RR = 1.21, 95% CI: 0.95–1.55, *P* = .124; *I^2^ = *69.9%, *P = *.068; Fig. 10). In addition, the pooled results show that the the total effective rate of acupuncture combined with TCM group was significantly higher than that in loratadine (RR = 1.31, 95% CI: 1.14–1.51, *P* ≤ .01; *I^2^ = *55.8%, *P = *.035) or cetirizine group (RR = 1.31, 95% CI: 1.19–1.44, *P* ≤ .01; *I^2^ = *35.1%, *P = *.187; Fig. 11). However, the pooled results show that there is no significant difference between acupuncture combined with TCM group and mizolastine group (RR = 1.21, 95% CI: 0.95–1.55, *P* = .124; *I^2^ = *69.9%, *P = *.068; Fig. 11).

### 3.5. Sensitivity analysis

We carried out sensitivity analysis by eliminating each included study one by one and performing a summary analysis of the remaining studies. Our research results found that none of the studies had an excessive impact on the results of the meta-analysis, which suggests that the results of this meta-analysis are stable and reliable. The results of the sensitivity analysis are shown in Figures S1 to S4 (Supplemental Digital Content, http://links.lww.com/MD/H178).

### 3.6. Publication bias

**Figure 12.**
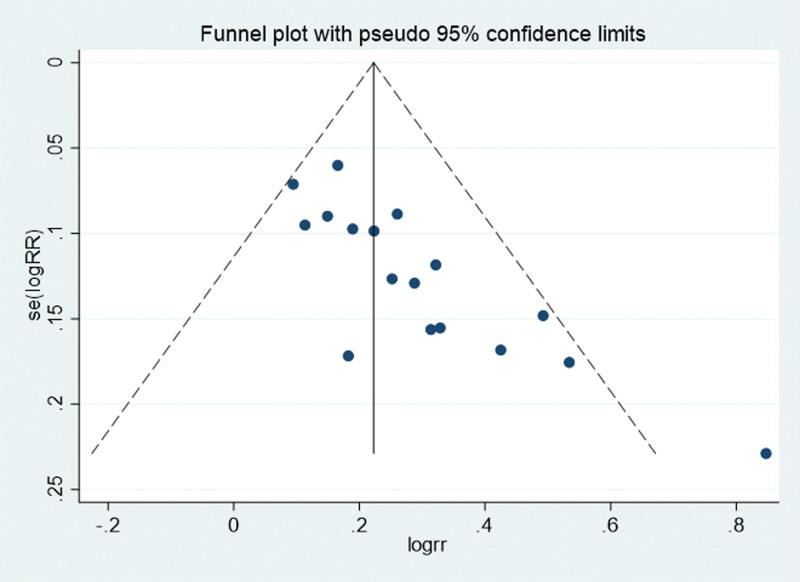
Funnel plot for evaluating the publication bias of this meta-analysis. RR = ratio risk.

The funnel plot of this study is shown in Figure 12. It can be seen that the funnel plot is asymmetric, and the *P* value of Egger test is ≤.01, indicating that there is a publication bias in this study.

## 4. Discussion

The Western medicine pathological mechanism of chronic urticaria is currently unclear, and it is mostly considered that the body’s allergic reaction under the action of special factors leads to local capillary congestion and expansion and inflammation. At present, Western medicine mostly uses antihistamines combined with immunosuppressive drugs or a combination of desensitizing drugs.^[26]^ Various clinical studies have expanded the treatment of chronic urticaria, but a radical cure has not yet been found, and there are problems with limited effects and high prices of some drugs.^[27]^ Therefore, more and more TCM treatments are also used in clinical practice. TCM physicians believe that the onset of chronic urticaria is related to external pathogens and internal causes and is considered to be caused by “wind evils” based on its onset characteristics of “occasionally appearing and appearing at different times, but with no fixed location.” These include exogenous wind evils, “outer wind” caused by the disharmony of camp and health, diet and emotional factors that cause liver-yang to transform wind and blood deficiency to generate wind. In addition to exogenous factors such as 6 evils, It is also closely related to insufficient body endowment and lack of righteousness. TCM syndrome differentiation is mostly based on the deficiency of the original and the essence of the original, the wind invades the body, and the deficiency of the qi cannot be fixed and the disease is caused.^[28]^ This Meta-analysis pooled 17 literature on the treatment of chronic urticaria with TCM combined with acupuncture, including 617 patients, which compares the efficacy and safety differences between acupuncture combined with conventional Western medicine, so as to provide guidance for the clinical treatment of chronic urticaria.

Our pooled results show that total effective rate of acupuncture combined with TCM group was significantly higher than that in conventional Western medicine group (RR = 1.29, 95% CI: 1.2–1.38, *P* ≤ .01). TCMs including Guizhi Decoction, Xiaofeng powder, etc, have been proven to be effective in clearing away heat and dampness, dispelling wind and nourishing blood. Dispelling wind and nourishing blood of TCM is the treatment of urticaria. Dispelling wind means to remove wind evil, and nourishing means to promote blood circulation. In the view of TCM, pruritus is caused by wind, humidity, and heat. In the treatment of heat to wind based, can drink anti-heat soup treatment. If it is chronic, in addition to being stimulated by wind, humidity, and heat, most of the wind to blood deficiency is related; blood stasis and qi stagnation is also inducement in the treatment to nourish blood to wind treatment.^[25]^ Acupuncture further promotes the efficacy of TCM and achieves the same treatment of symptoms and root causes. Further subgroup analysis results show the total effective rate of acupuncture combined with Guizhi Decoction (RR = 1.44, 95% CI: 1.15–1.81) or Xiaofeng powder group (RR = 1.31, 95% CI: 1.16–1.47) was significantly higher than that in conventional Western medicine group. However, there is no significant difference between acupuncture combined with Yangxue Xiaofeng Decoction group and conventional Western medicine group. This result suggests that Guizhi Decoction and Xiaofeng powder combined with acupuncture can achieve significant effects in the treatment of chronic urticaria. In addition, according to the RR value, it can be guessed that Guizhi Decoction may have a greater effect than Xiaofeng powder. However, this inference currently lacks the results of the difference test. Moreover, as this meta-analysis included only 2 articles that reported Yangxue Xiaofeng Decoction, the results may lack objectivity, and more clinical trials are needed to verify its efficacy. In addition, we found that the total effective rate of acupuncture combined with TCM group was significantly higher than that in loratadine or cetirizine group. However, there is no significant difference between acupuncture combined with TCM group and mizolastine group. This result may inspire us, if we want to add western medicine on the basis of Chinese medicine combined with acupuncture in the treatment of chronic urticaria, mizolastine may be a better choice.

We also analyzed the degree of improvement in clinical symptoms. The pooled results show that UAS and pruritus score of acupuncture combined with TCM group was significantly lower than that in conventional Western medicine group. However, there is no significant difference between acupuncture combined with TCM group and conventional Western medicine group. UAS is a comprehensive score based on the size of the wind mass and the degree of itching. The results of this study show that the combination of acupuncture and medicine has a significant effect in improving the syndrome. The results of this study show that the combination of acupuncture and medicine has a significant effect in improving the syndrome. It is worth noting that the combination of acupuncture and medicine is more conducive to alleviating the degree of itching.

Importantly, our pooled results also show that recurrence rate and the incidence of adverse events of acupuncture combined with TCM group were all significantly lower than that in conventional Western medicine group. This further shows that acupuncture and TCM can treat chronic urticaria in concert, which can promote the effect of TCM, achieve the same treatment of symptoms and root causes, and reduce the recurrence rate and the incidence of adverse reactions. At the same time, it has increased our confidence in the treatment of chronic urticaria with TCM. We should further promote the application of TCM combined with acupuncture in the treatment of chronic urticaria.

This meta-analysis also has the following shortcomings: There is heterogeneity in the study. Our subgroup analysis for different Chinese and Western medicines did not exclude the heterogeneity, indicating that the heterogeneity may be due to differences in the patient’s condition; The publication bias results show that there is publication bias in this study, which may reduce the objectivity of the results of this study. In the future, more updated studies need to be included to avoid publication bias and further verify the results of this study.

## 5. Conclusion

Our research results found that TCM combined with acupuncture has a more significant effect than conventional Western medicine and can significantly reduce the recurrence rate and the incidence of adverse reactions. The application of TCM combined with acupuncture in the treatment of chronic urticaria should be further promoted.

## Author contributions

Zhenxiong Lu conceived the study and wrote the manuscript. Qiujun Zhou, Shiqian Chai, Huifeng Yang, and Jinhui Wang participated in data collection. Maocan Tao and Hongbin Luo participated in data analysis. Yi Cao and Hongbin Luo conceived the final approval of the version to be submitted and obtaining of funding.

## Supplementary Material


