# Supplementary material for: Efficacy and safety of Chinese medicine combined with acupuncture in the treatment of chronic urticaria: A meta-analysis
Source: Medicine (Baltimore). 2022 Sep 9;101(36):e30381. doi: 10.1097/MD.0000000000030381 (PMC10980365; doi:10.1097/MD.0000000000030381)
Supplement: Supplementary file 1 [file medi-101-e30381-s001.pdf]

Sensitivity analysis

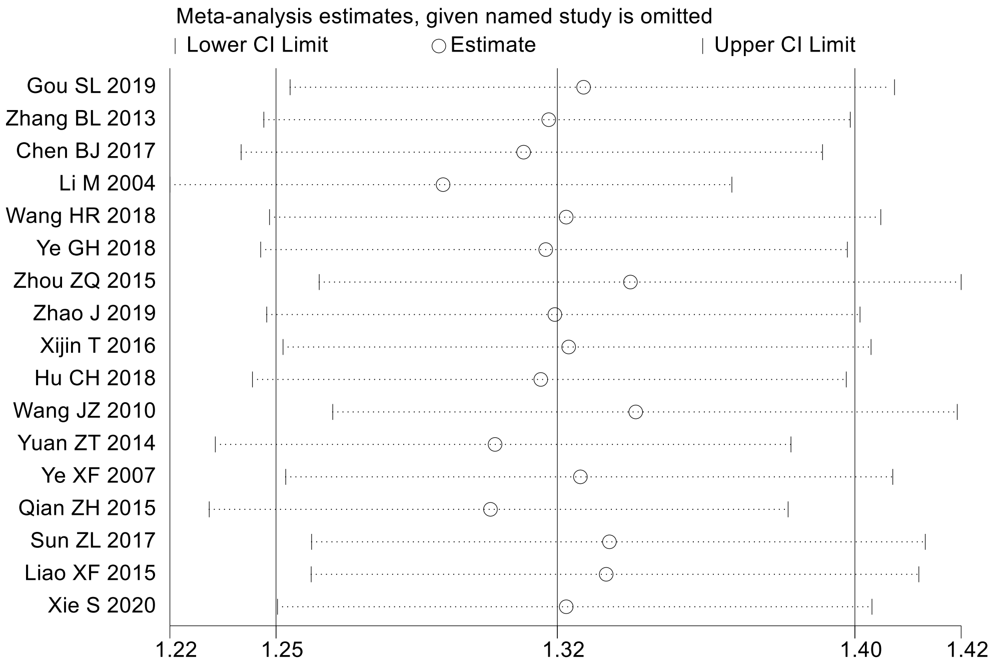

Figure S1. Total effective rate of acupuncture combined with traditional Chinese medicine group and conventional Western medicine group

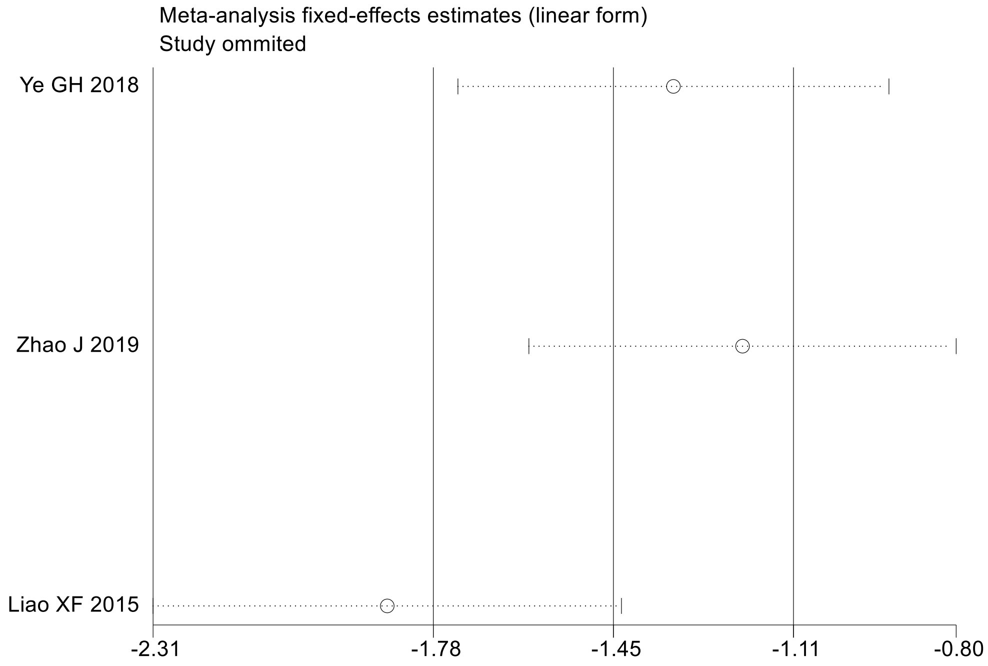

Figure S2. UAS of acupuncture combined with traditional Chinese medicine group

and conventional Western medicine group

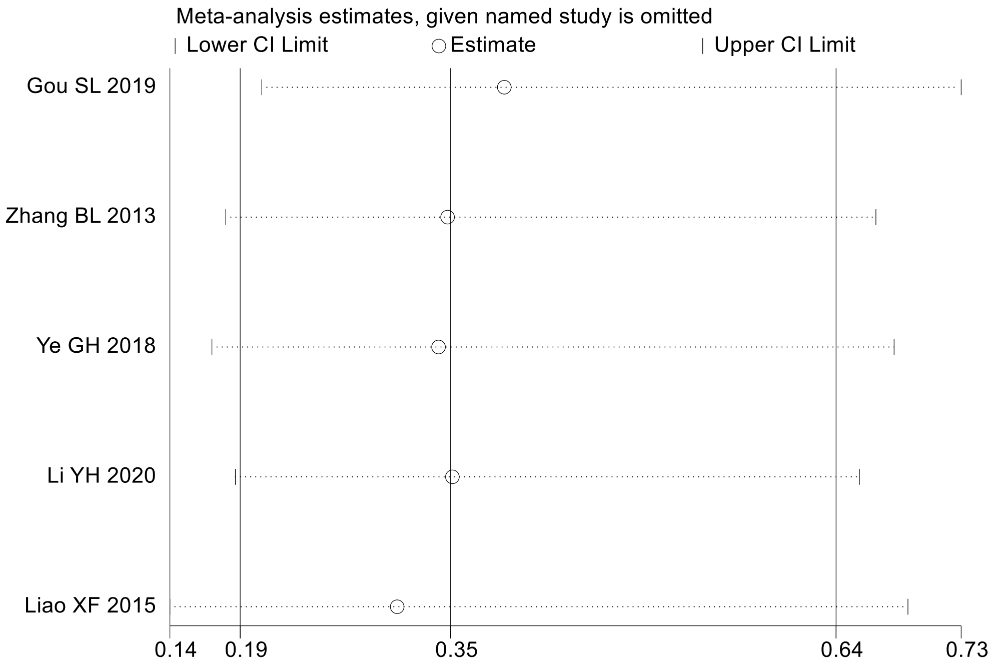

Figure S3. Recurrence rate of acupuncture combined with traditional Chinese medicine group and conventional Western medicine group

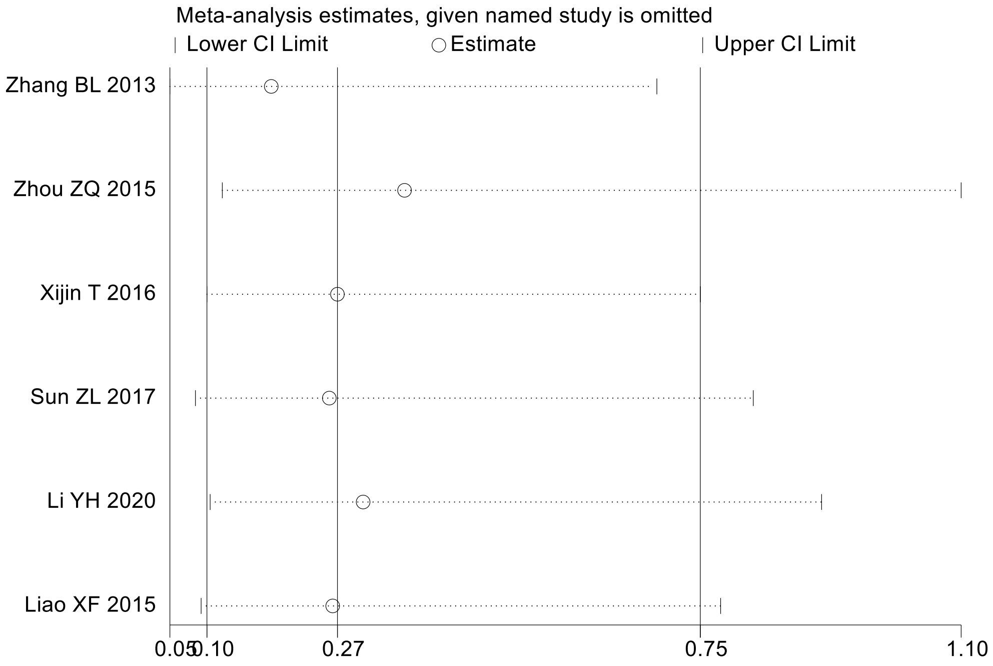

Figure S4. Incidence of adverse events of acupuncture combined with traditional

Chinese medicine group and conventional Western medicine group
